# Supplementary material for: Psychophysical reverse correlation reflects both sensory and decision-making processes
Source: Nat Commun. 2018 Aug 28;9:3479. doi: 10.1038/s41467-018-05797-y (PMC6113286; doi:10.1038/s41467-018-05797-y)
Supplement: Supplementary file 1 — Supplementary Information [file 41467_2018_5797_MOESM1_ESM.pdf]

Supplementary Information for

Psychophysical reverse correlation reflects both sensory and decision-making processes

Gouki Okazawa[1], Long Sha[1], Braden A. Purcell[1], Roozbeh Kiani[1,2,3]

[1] Center for Neural Science, New York University, New York, NY 10003
[2] Department of Psychology, New York University, New York, NY 10003
[3] Neuroscience Institute, NYU Langone Medical Center, New York, NY 10016

Corresponding Author:
Roozbeh Kiani
Center for Neural Science
New York University
4 Washington Pl, Room 809, New York, NY 10003
roozbeh@nyu.edu

Supplementary Note 1

Here we expand the Results section on "Speed-accuracy tradeoff, bias, and more complex decision models" to provide better intuition and mechanistic explanations for kernel dynamics caused by different parameters of competing accumulator models.

Effect of input correlation of competing accumulators on psychophysical kernels: Sizeable distortions arise only when the input correlation approaches 0, in which case the kernel is initially inflated but later drops below the true sensory weight (Fig. 6e and Supplementary Fig. S8a). The inflation is caused by an effective increase in the diffusion noise because for low input correlations the noise in either integrator can facilitate a bound crossing. However, as time passes, the mean DV of unterminated decisions becomes increasingly more negative due to diffusion noise and attrition of trials whose DV exceeds the bound. The more negative DVs reduce the effect of new input for determining the outcome of the process, shrinking the kernel below the true sensory weights.

Effect of lower reflective bound on psychophysical kernels: Unlike the input correlation, reflective bounds cause the psychophysical kernel to begin lower than the true sensory weight but exceed it later (Fig. 6g-i and Supplementary Fig. 8b). When the reflective bounds are far enough from the starting point of the integrators, they tend to have only a modest effect on the psychophysical kernel. However, their effect grows quickly as the reflective bound approaches the starting point. The initial underestimation happens because reflective bounds limit movements below the starting point and, thus, reduce both the effective noise and the effective counter-evidence for each choice. Later in the integration process, the integrators are on average closer to their decision bounds compared to a model without lower reflective bounds. This amplifies the psychophysical kernel because input fluctuations are more likely to lead to a decision bound crossing.

Effect of mutual inhibition on psychophysical kernels: Mutual inhibition amplifies the difference of the two integrators and effectively prevents the losing integrator from gaining the upper hand[1-3]. This suppression can cause dramatic distortions in psychophysical kernels, especially early in the integration process, because the mutual inhibition magnifies the effect of early sensory evidence on the state of the two integrators (Fig. 6j-l brown lines and Supplementary Fig. 8c). As the integration process continues and the losing integrator drops far enough from its decision bound to exert significant inhibition on the other integrator, the behavior of the model converges to a simple DDM and the kernel converges on the true sensory weights.

Effect of leak and its balance with mutual inhibition on psychophysical kernels: When mutual inhibition dominates (leak/inhibition ratio<1), psychophysical kernels show an early amplification but later converge on the true sensory weights (Fig. 6k, red lines), for the same reasons explained in the previous paragraph. When leak and inhibition balance each other out, the model acts similar to a line attractor and the psychophysical kernels resemble those of a DDM (Fig. 6k, black lines). Finally, when leak dominates, the integrators lose information and decisions are influenced less by input fluctuations, especially for early sensory evidence in the trial. Consequently, stimulus-aligned psychophysical kernels systematically underestimate the sensory weights (Fig. 6k, blue lines). However, the dynamics of the kernel qualitatively resemble the true sensory weight, except for the earliest times. On the other hand, the response-aligned kernels are distorted and accelerated compared to a DDM, reflecting the shorter integration time constant and stronger influence of later evidence on the decision[4,5].

Supplementary Discussion

To maximize the information gained from psychophysical kernels, it is important to set up the model fitting and model prediction in such a way that minimizes overlap between the fitted and predicted aspects of the data. For example, using stimulus fluctuations on individual trials to predict the choice and calculating model kernels for the same stimulus fluctuations are unlikely to provide new insights because any model that fits the choices well could also replicate the kernels. However, by leaving the specific stimulus fluctuations aside for fitting the choices or by predicting kernels for a non-overlapping group of trials one can reveal potential discrepancies between the model and data.

A key piece of information for proper interpretation of psychophysical kernels is to know which part of the stimulus is used for the decision-making process. Fixed duration tasks with long stimulus durations are generally unsuitable because the start and termination times of the decision-making process are opaque to the experimenter. When subjects commit to a choice by integration of sensory evidence toward a bound, they tend to ignore later evidence, causing a downward trend in the kernels. However, if the integration process also begins at variable times, the downward trend can be masked. Overall, the presence or absence of temporal dynamics in psychophysical kernels in fixed-duration tasks does not have a unique interpretation. It is more suitable to use tasks in which stimulus duration is controlled by the experimenter and varies randomly from trial to trial because they enable the experimenter to determine which part of the stimulus is used for the decision. However, one should be careful in using those designs because just the variability of stimulus duration in itself can introduce temporal dynamics in kernels (Eq. 14). Reaction time task designs are most suitable because they minimize ambiguity about which part of the stimulus was used for the decision-making process.

We also note that tasks that use very brief stimulus presentations are not immune to the influence of the decision-making process on psychophysical kernels. Brief stimulus presentations are often used to infer the spatial structure of sensory filters at the cost of ignoring their temporal dynamics. However, brief stimulus presentations do not guarantee instantaneous decisions. Several studies have demonstrated that accumulation of evidence to a bound is at work even for brief stimuli[6,7], as evidenced by large RT differences for different stimulus strengths. Even brief stimulus presentations produce extended trails of activity in sensory and action-planning neurons[8-10]. An extended decision-making process for a briefly presented stimulus makes the kernel susceptible to the deviations explained above. For example, a change in speed-accuracy tradeoff can amplify the inferred spatial filters without a real change in sensory processes.

Using a fixed-duration design, several studies have found monotonically decreasing psychophysical kernels[5,11,12]. Kernel dynamics in those studies could have a sensory origin or be due to static sensory weights and a decision-making mechanism that terminates according to some criteria (e.g., Fig. 3g). It has been common to assume one possibility and ignore the other. Testing these two possibilities explicitly is likely to yield new insights and trends across experiments. Several other studies have reported a flat psychophysical kernel in fixed-duration designs and interpreted it as a signature of perfect integration of sensory evidence. This interpretation could be correct if subjects set their decision bound too high to reach during stimulus viewing, as shown in Brunton et al.[13]. However, a static kernel could also arise from a variety of sensory and decision-making mechanisms and does not uniquely support perfect integration of sensory evidence. Also, in addition to the mechanisms explained in Results, a static kernel in a non-RT task could be produced with probabilistic sampling of evidence rather than its integration. In general, it is best not to rely solely on qualitative patterns of psychophysical kernels. As we suggest in this paper, these qualitative signatures should be just a starting point for building

mechanistic hypotheses, which should then be tested with detailed, quantitative modeling of behavior and electrophysiological studies of its underlying neural responses.

Supplementary Figures

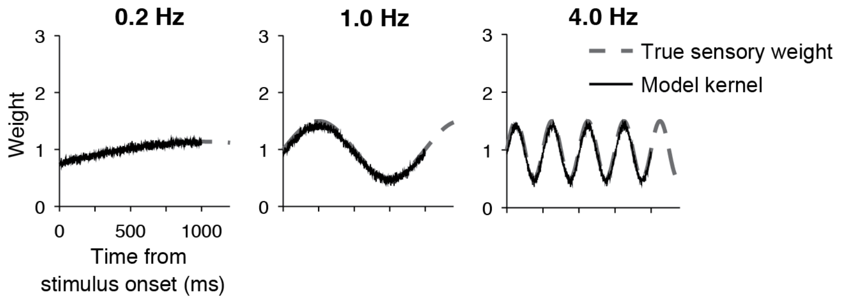

Supplementary Figure 1. For an unbounded DDM, psychophysical kernels recover the true sensory weights. Related to Fig. 3a-b. This figure shows three simulated models with sensory weights that fluctuated at 0.2, 1, and 4 Hz. Any frequencies of weights could be accurately recovered by the psychophysical kernel. See Fig. 3c for the quantification of kernel distortions as a function of temporal frequency of weights.

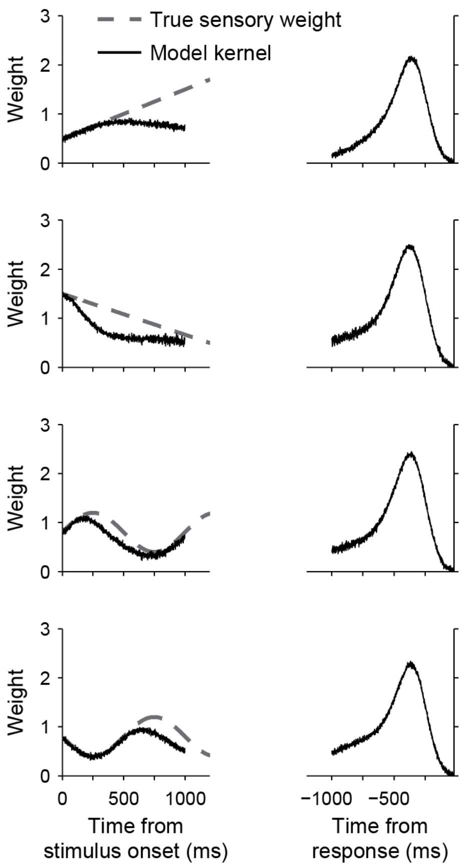

Supplementary Figure 2. Psychophysical kernels deviate from sensory weights because of incomplete knowledge about decision time. Related to Fig. 3i-m. The figure shows four simulated bounded DDMs with non-decision time and time varying sensory weights. The mean and standard deviation of non-decision time are set to 300ms and 100ms, respectively, and decision bound is set to 30. The non-decision time causes the psychophysical kernels to systematically underestimate the true sensory weights.

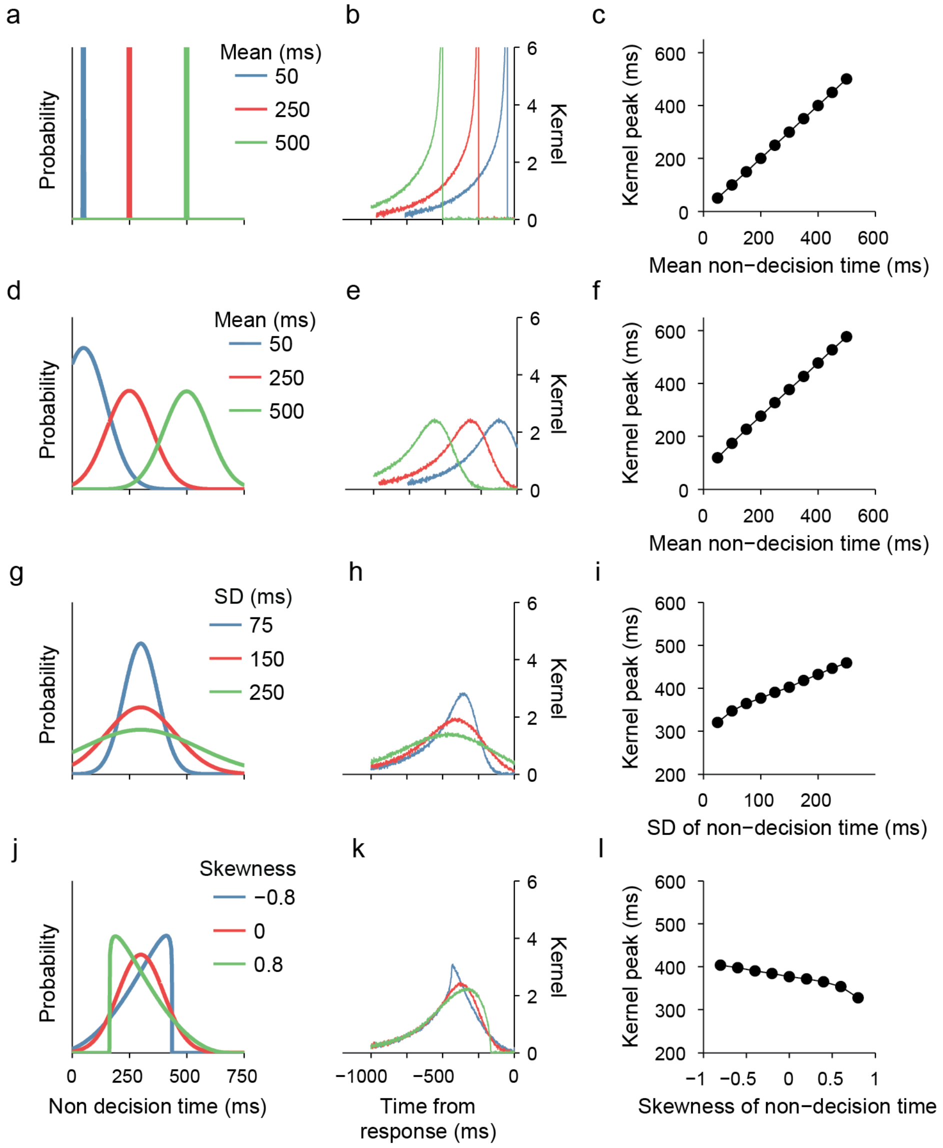

Supplementary Figure 3. The distribution of non-decision times determines the shape of response-aligned psychophysical kernels. Sensory and motor delays in a RT task cause the stimulus fluctuations immediately prior to the response not to bear on the choice. As a result, response-aligned kernels tend to show a peak before the behavioral response (Figs. 3-5). The time and shape of this peak are informative about the distribution of non-decision times. Here, we simulate DDMs with non-decision time distributions that vary in mean, variance, and skewness. Each row illustrates three sample distributions (left column), their corresponding kernels (middle column), and systematic effects on peak time as the non-decision time distribution changes (right column). (**a-c**) For a constant non-decision time, kernels show an abrupt drop before the response. The temporal gap between this drop and response is identical to the non-decision time. (**d-f**) When non-decision time is variable, changes in mean non-decision time shifts the kernel peak time with minimal changes in its shape. Standard deviation and skewness of the distributions are set to 100 and 0, respectively, in these simulations. (**g-i**) Larger variance of non-decision time widens the kernel and shifts its peak away from the response. Mean and skewness of non-decision time are set to 300ms and 0, respectively. (**j-l**) Skewness of non-decision time creates asymmetries in the peak. More positive skewness pushes the peak toward the response. Mean and standard deviation of non-decision time are set to 300ms and 100ms, respectively. In all simulations the sensory weight function is static ($w(t)=1$) and decision bound is set to 30.

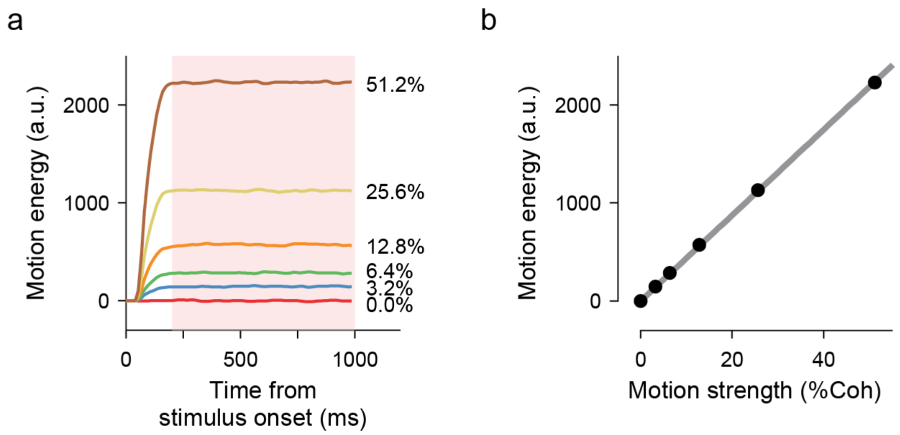

Supplementary Figure 4. Mean motion energy is a linear function of net motion strength (coherence). (**a**) Time course of average motion energy for different coherence levels in the direction discrimination task. To calculate the scaling between motion coherence and motion energy, we computed the average motion energy 200-1000ms after stimulus onset (pink rectangle). (**b**) Average motion energy as a function of motion coherence.

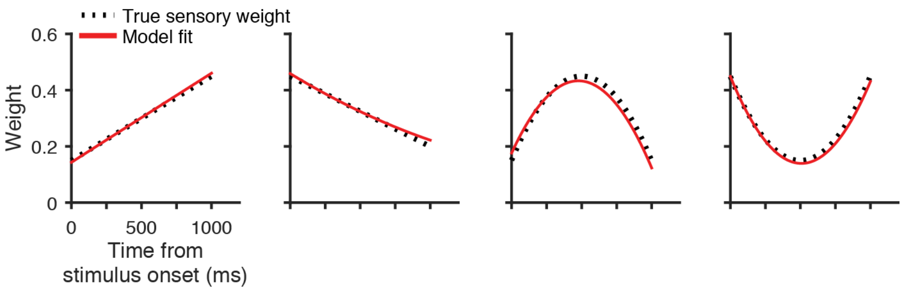

Supplementary Figure 5. An extended DDM can recover changes of sensory weights, when such changes are present. The figure shows four simulations with different weight dynamics and the recovered weights of the model. For each panel, we simulated a direction discrimination dataset with 5000 trials. Like the real task explained in the paper, motion strength on each trial was selected randomly from a fixed set (0%, 3.2%, 6.4%, 12.8% or 25.6%). The weights in each trial could change according to a second order polynomial function. Simulated sensory evidence at each moment in a trial was a random draw from a Gaussian distribution with s.d. = 1 and mean = $w(\tau)s$, where s is the motion coherence on the trial. Momentary evidence was accumulated until a positive or negative bound was reached (bound height, 30). The bound dictated the choice and time to bound was decision time. Reaction time was the sum of decision time and a random non-decision time drawn from a Gaussian distribution with mean = 300ms and s.d. = 100ms. We fit the extended DDM model with polynomial weight dynamics (Eq. 23 in Methods) to the distribution of choices and RTs of the 5000 simulated trials in each panel.

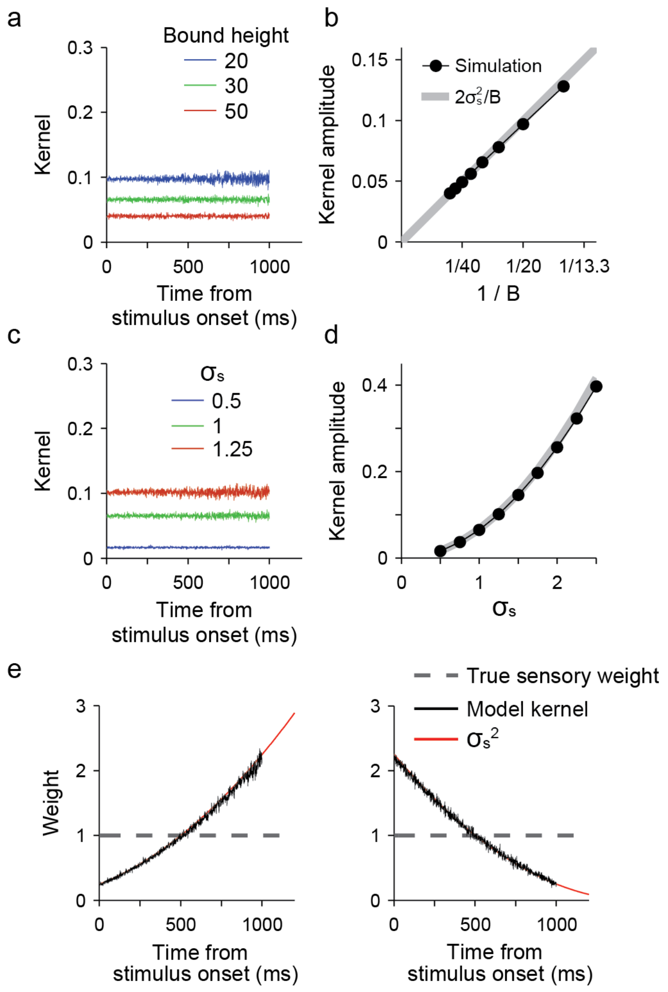

Supplementary Figure 6. Scaling of psychophysical kernels by bound height and stimulus noise in a bounded DDM. As we prove in Methods, the psychophysical kernel of a bounded DDM without non-decision time is proportional to the sensory weight and the constant of proportionality is $\frac{2\sigma_s^2}{B}$ (Eq. 2 in Results). Here we confirm this relationship by simulating the model. (**a-b**) Higher decision bounds produce lower amplitudes of psychophysical kernel (a). The mean amplitude of the kernel is proportional to the inverse of decision bound (b). The gray line shows expected kernel amplitude based on Eq. 2 and the black lines and points show simulated amplitudes. σ_s^2 is set to 1 in these simulations. (**c-d**) Higher standard deviation of stimulus fluctuation raises the amplitude of psychophysical kernel (c). The kernel amplitude is a quadratic function of σ_s (d). B is set to 30 for these simulations. **(e)** When stimulus noise (σ_s) changes within the trial, the psychophysical kernel co-varies with it, causing deviation from the true sensory weight. Note that in Figs. 3, 6, 7, Supplementary Figs. 1-2, and 7-9, we normalized the kernel based on Eq. 2 and Eq. 14 to remove the effect of bound height and stimulus variance in order to compare the kernel directly with the sensory weight.

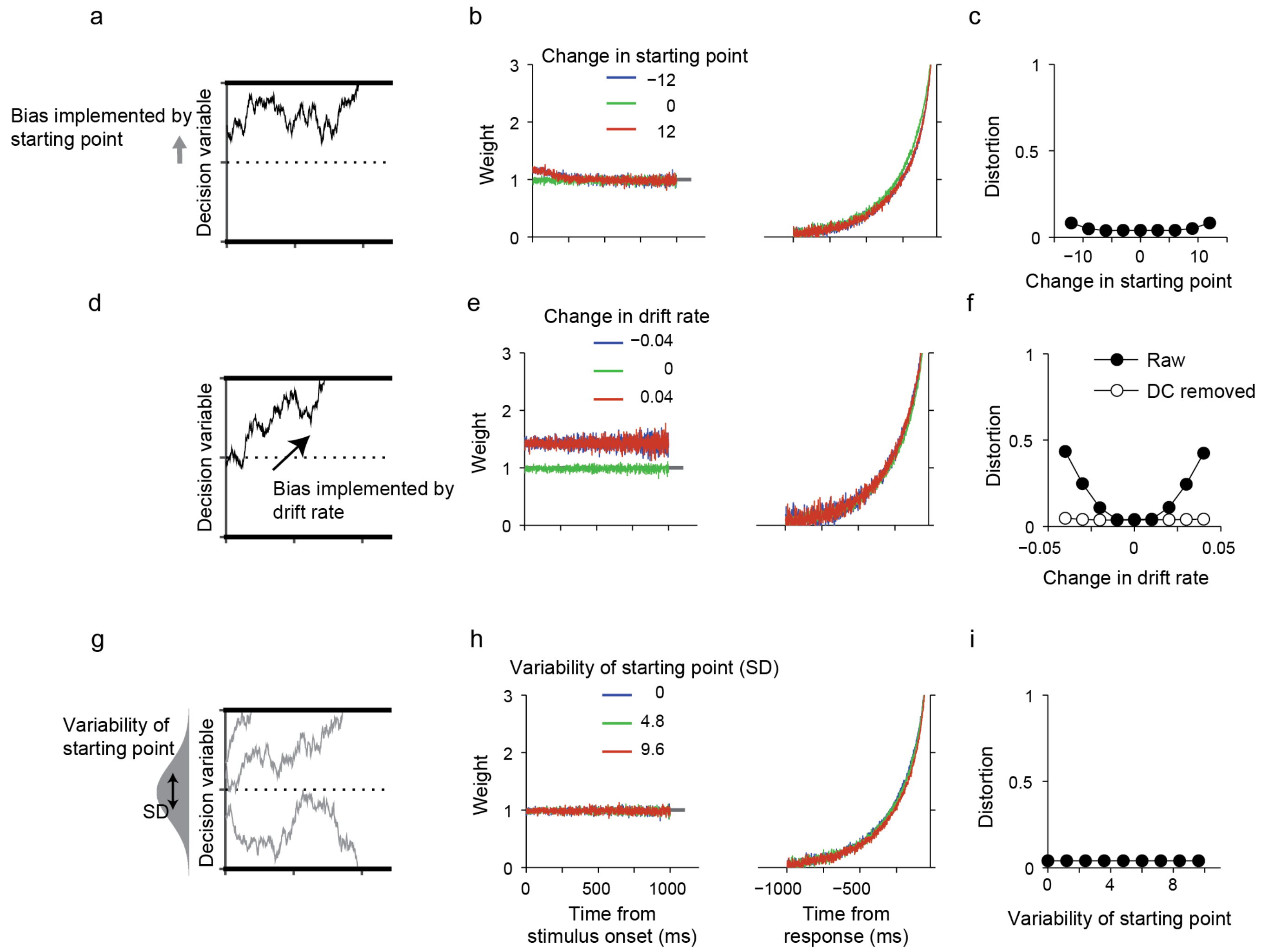

Supplementary Figure 7. The effect of bias and variability of the starting point of the DDM on psychophysical kernels. Conventions are similar to Fig. 6, where the first column shows the model variation, the second column shows example parameterizations of the model, and the third column shows the magnitude of distortion of the psychophysical kernel as a function of the parameter of interest. Decision bound is set to 30 in all simulations. (**a-c**) When choice bias is implemented by a shift of the starting point toward a decision bound, the kernel shows a small inflation around stimulus onset, because the closer distance of the starting point to one of the bounds increases the likelihood of bound crossing due to early stimulus fluctuations. (**d-f**) When choice bias is implemented by a constant change in drift rate, the kernel shows a DC offset. (**g-i**) Trial-to-trial variability of the starting point of the DDM does not cause a systematic distortion of psychophysical kernels, if the starting point distribution is centered on zero.

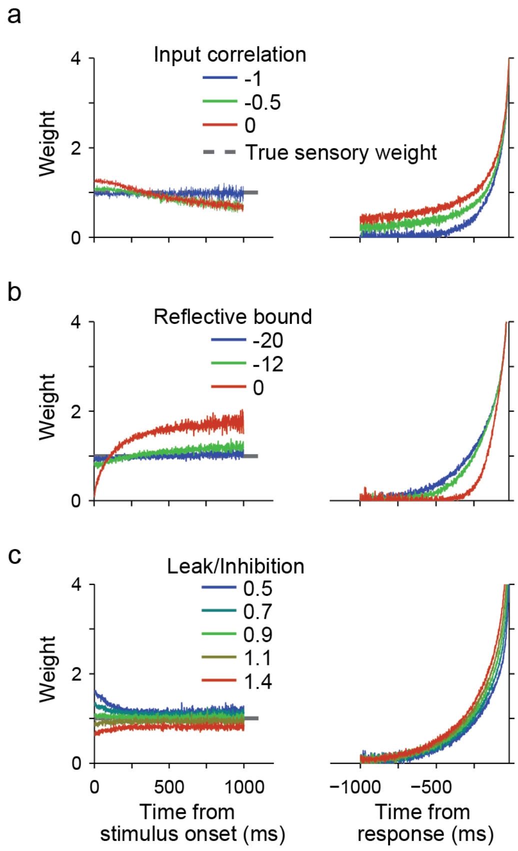

Supplementary Figure 8. Additional examples of psychophysical kernels for various parameterizations of the competing accumulator model. To provide better intuition for the effects of model parameters in Fig. 6, we have plotted psychophysical kernels for multiple parameter values. a-d correspond to rows 2-4 in Fig. 6.

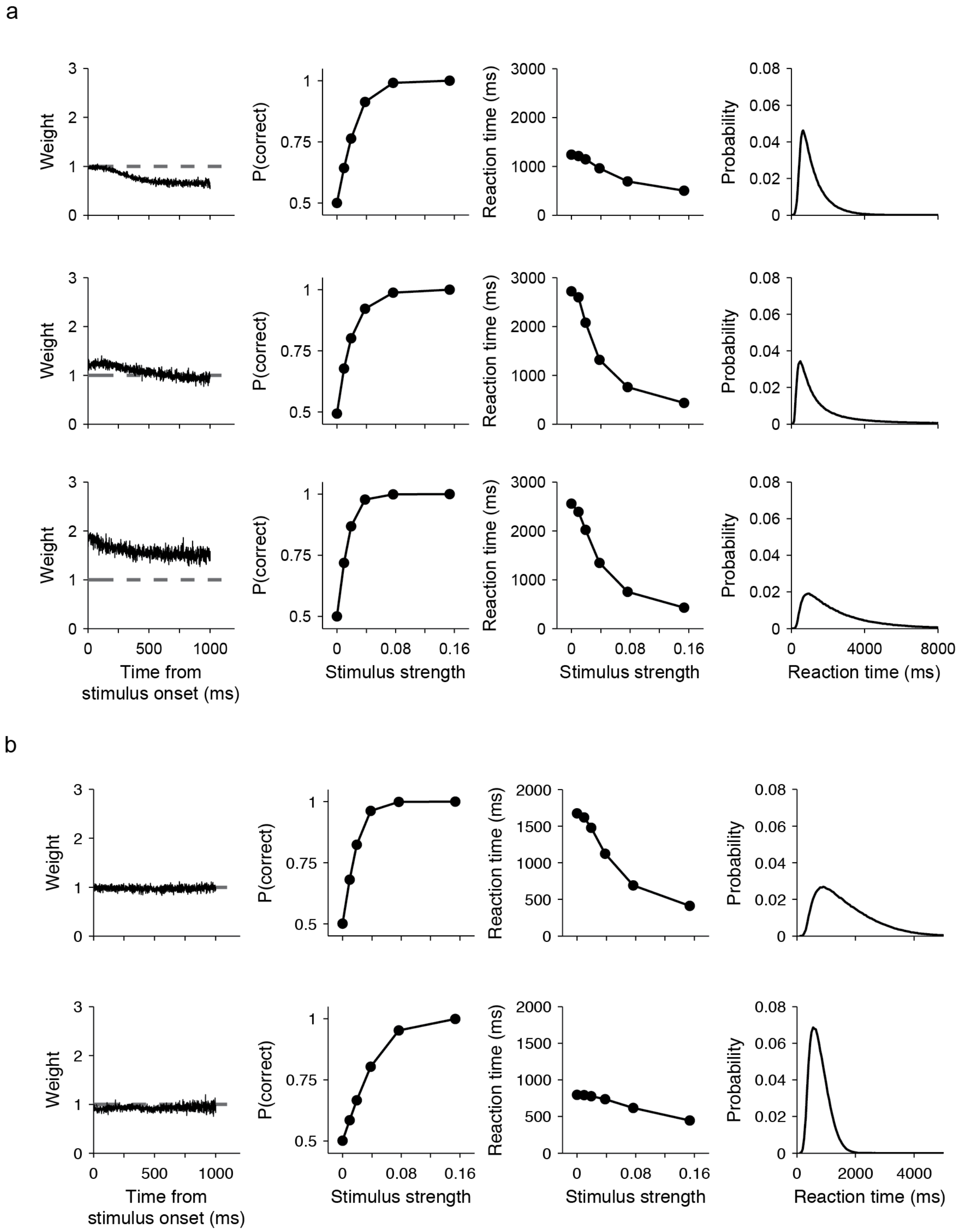

Supplementary Figure 9. A three-pronged approach based on the shape of psychophysical kernels and the distribution of choices and RTs can distinguish different mechanisms that contribute to the decision-making process. (**a**) A diversity of mechanisms can lead to similar trends in psychophysical kernels but they usually lead to contrasting psychometric and chronometric functions, different RT distributions, and quantitative differences in the shape of kernels. The figure shows three mechanisms that cause a downward trend in stimulus-aligned kernels. The top row shows a DDM with mean non-decision time set to 300ms and s.d. of non-decision time to 100ms (B = 30). The middle row shows a competing accumulator model where the input correlation of the two accumulators is −0.1 (Leak and inhibition are set to 0; non-decision time, mean, 100ms, s.d., 33ms; B = 50). Bottom row shows a competing

accumulator model where the leak to inhibition ratio is 0.8 (L, 0.0027; I, 0.0033; input correlation, −1; non-decision time, mean, 100ms, s.d., 33ms; B = 80, $v_0 = 30$). RT distributions in the right column are for trials with stimulus strength of 0, the same trials used for making the psychophysical kernels. The parameters of the three simulations are adjusted to have a more or less similar drop in psychophysical kernels. Kernels are normalized according to Eq. 2 in the main text. (**b**) A flat psychophysical kernel can emerge from a diversity of mechanisms, which often cause contrasting psychometric and chronometric functions, and different RT distributions. Top row shows a DDM with urgency ($\tau_{1/2}$, 5,000ms, b, 50, u_∞, 50 in Eq. 15; non-decision time, mean, 100ms, s.d., 33ms). Bottom row show the competing accumulator model of Fig. 7d.

Supplementary References

1 Wang, X.-J. Probabilistic decision making by slow reverberation in cortical circuits. *Neuron* **36**, 955-968 (2002).

2 Wong, K.-F. & Wang, X.-J. A recurrent network mechanism of time integration in perceptual decisions. *Journal of Neuroscience* **26**, 1314-1328 (2006).

3 Albantakis, L. & Deco, G. Changes of mind in an attractor network of decision-making. *PLoS Computational Biology* **7**, e1002086 (2011).

4 Usher, M. & McClelland, J. L. The time course of perceptual choice: the leaky, competing accumulator model. *Psychological Review* **108**, 550-592 (2001).

5 Kiani, R., Hanks, T. D. & Shadlen, M. N. Bounded integration in parietal cortex underlies decisions even when viewing duration is dictated by the environment. *Journal of Neuroscience* **28**, 3017-3029 (2008).

6 Ratcliff, R. & Rouder, J. N. A diffusion model account of masking in two-choice letter identification. *Journal of Experimental Psychology. Human Perception and Performance* **26**, 127-140 (2000).

7 Smith, P. L. & Ratcliff, R. An integrated theory of attention and decision making in visual signal detection. *Psychological Review* **116**, 283-317 (2009).

8 Kira, S., Yang, T. & Shadlen, M. N. A neural implementation of Wald's sequential probability ratio test. *Neuron* **85**, 861-873 (2015).

9 Zhou, J., Benson, N. C., Kay, K. N. & Winawer, J. Compressive temporal summation in human visual cortex. *Journal of Neuroscience* **38**, 691-709 (2018).

10 Kovács, G., Vogels, R. & Orban, G. A. Cortical correlate of pattern backward masking. *Proceedings of the National Academy of Sciences, USA* **92**, 5587-5591 (1995).

11 Mareschal, I., Dakin, S. C. & Bex, P. J. Dynamic properties of orientation discrimination assessed by using classification images. *Proceedings of the National Academy of Sciences, USA* **103**, 5131-5136 (2006).

12 Nienborg, H. & Cumming, B. G. Decision-related activity in sensory neurons reflects more than a neuron's causal effect. *Nature* **459**, 89-92 (2009).

13 Brunton, B. W., Botvinick, M. M. & Brody, C. D. Rats and humans can optimally accumulate evidence for decision-making. *Science* **340**, 95-98 (2013).
